# Identification of a Novel SBP1-Containing SCF^SFB^ Complex in Wild Dwarf Almond (*Prunus tenella*)

**DOI:** 10.3389/fgene.2019.01019

**Published:** 2019-10-25

**Authors:** Bin Zeng, Jianyou Wang, Qing Hao, Zhenfan Yu, Ayimaiti Abudukayoumu, Yilian Tang, Xiangfei Zhang, Xinxin Ma

**Affiliations:** ^1^College of Forestry and Horticulture, Xinjiang Agricultural University, Urumqi, China; ^2^Department of Crop Genetics and Breeding, Sub-branch of National Melon and Fruit Improvement Centre, Urumqi, China; ^3^Department of Horticultural Crops, Xinjiang Branch of China Academy of Forestry Sciences, Urumqi, China; ^4^Institute of Horticultural Crops, Xinjiang Academy of Agricultural Sciences, Urumqi, China

**Keywords:** dwarf almond, *SBP1*, SCF (SKP1–Cullin1–F-box-Rbx1) complex, self-incompatibility, *Prunus*

## Abstract

S-RNase-based gametophytic self-incompatibility (SI), in which specificities of pistil and pollen are determined by S-RNase and the S locus F-box protein, respectively, has been discovered in the Solanaceae, Plantaginaceae, and Rosaceae families, but some underlying molecular mechanisms remain elusive and controversial. Previous studies discovered SI in wild dwarf almond (*Prunus tenella*), and pistil S (S-RNase) and pollen S (SFB) determinant genes have been investigated. However, the SCF (SKP1–Cullin1–F-box-Rbx1) complex, which serves as an E3 ubiquitin ligase on non-self S-RNase, has not been investigated. In the current study, *PetSSK1* (*SLF-interacting-SKP1-like1*), *SBP1* (*S-RNase binding protein 1*), *CUL1*, and *SFB* genes (S-haplotype-specific F-box) were identified in an accession (ZB1) of *P. tenella*. Yeast two-hybrid assays revealed interactions between *PetSBP1* and *PetCUL1* and between *PetSBP1* and *PetSFB*s (*SFB16* and *SFB17*), and subsequent pull-down assays confirmed these interactions, suggesting a novel SBP1-containing SCF^SFB^ complex in wild dwarf almond. Moreover, despite a putative interaction between *PetSSK1* and *PetCUL1*, we revealed that *PetSSK1* does not interact with *PetSFB16* or *PetSFB17*, and thus the canonical SSK1-containing SCF^SFB^ complex could not be identified. This suggests a novel molecular mechanism of gametophytic SI in *Prunus* species.

## Introduction

Mate choice is an essential process during sexual reproduction of plant species. Self-incompatibility (SI), a widespread reproductive barrier in multiple flowering plants, can inhibit self-fertilization and promote out-crossing by denying self (genetically related) pollen while accepting non-self (genetically unrelated) pollen for fertilization (Matsumoto et al., 2012). SI is common among angiosperms and could be found in approximately 60% of angiosperm species, in at least 19 orders, 71 families, and 250 genera (Akagi et al., 2016). SI plays important roles in the diversification and differentiation of species by preserving genetic diversity. There are two genetically distinct types of SI: gametophytic SI (GSI) and sporophytic SI (SSI), which were distinguished by how the incompatibility phenotype of the pollen is resolved (Hiscock and Tabah, 2003).

An S-RNase-based GSI system in which pistil-part specificity is dominated by an extracellular cytotoxic ribonuclease, S-RNase, encoded by the highly polymorphic S locus, has been discovered in the Solanaceae, Plantaginaceae, and Rosaceae families (Lai et al., 2002; Sijacic et al., 2004). Phylogenetic analyses of S-RNase and related sequences indicated that the S-RNase-based GSI system originated independently about 120 million years ago (Igic and Kohn, 2001; Steinbachs and Holsinger, 2002; Vieira et al., 2008). If this system is lost in species, it can never be regained (Igic et al., 2006; Goldberg et al., 2010; Igic and Busch, 2013).

The SCF (SKP1–Cullin1–F-box-Rbx1) complex, which consists of SKP1 (S-phase kinase-associated protein 1), F-box, CUL1, and Rbx1 (Moon et al., 2004), is a core factor in S-RNase-based GSI and serves as an E3 ubiquitin ligase on non-self S-RNase (Hua et al., 2008). F-box protein encoded by *SLF* (*S-locus F-box*) in Solanaceae and Plantaginaceae and *SFB* (*S-haplotype-specific F-box*) in Rosaceae (Chen et al., 2010) binds to S-RNase specifically, causing ubiquitin degradation of S-RNase (Tyers and Jorgensen, 2000). SKP1 functions as an adaptor to connect the variable F-box protein to CUL1, and CUL1 forms a core catalytic scaffold with Rbx1 (Zheng et al., 2002; Deshaies and Joazeiro, 2009).

SSK1 (SLF-interacting SKP1-like1), a pollen-specific protein, can bind to SLF/SFB and CUL1 to form the SCF complex participating in the ubiquitin reaction of S-RNase in petunia (*Petunia hybrida*) (Zhao et al., 2010), antirrhinum (*Antirrhinum hispanicum*) (Huang et al., 2006), apple (*Malus domestica*) (Yuan et al., 2014), sweet cherry (*Prunus avium*) (Matsumoto et al., 2012), and pear (*Pyrusbrets chneideri*) (Xu et al., 2013). The ubiquitously expressed protein SBP1 (S-RNase Binding Protein 1), which can bind to the hypervariable region of S-RNase (Sims and Ordanic, 2001; Hua and Kao, 2006), was first found to functionally adopt the function of SKP1 and Rbx1 in bridging CUL1 and the F-box, to form the SCF complex in *Petunia inflata* (Hua and Kao, 2006), and functions as an E3 ubiquitin ligase in recognizing and degrading S-RNase *in vitro* (Hua and Kao, 2008). Both SSK1 and SBP1 were found to be components of the SCF complex in petunia (Sims et al., 2010), while non-canonical SBP1-containing and canonical SBP1-containing SCF complexes were identified in apple (Minamikawa et al., 2014). There is currently no other evidence demonstrating that SBP1 is one of the components of the SCF complex, including in *Prunus* species.

The wild dwarf almond (*Prunus tenella* syn. *Amygdalus nana*) is discovered in small, isolated populations on the Balkan Peninsula, and GSI has been established by pollination experiments (Surbanovski et al., 2007). In recent years, some small and isolated populations have also been discovered scattered throughout the Xinjiang Uygur Autonomous Region of China (**Figure S1** and **Table S1**). As a species related to *P. avium*, *P. tenella* was speculated to have a similar SI system. After successful molecular identification of *CUL1*, *SSK1*, *SBP1*, and *SFBs* in an accession of *P. tenella*, we carried out yeast two-hybrid (Y2H) and pull-down experiments. To our surprise, a non-canonical SBP1-containing rather than a canonical SSK1-containing SCF^SFB^ complex was identified.

## Result

### Identification of Components of the SCF Complex

The putative canonical SSK1-containing SCF^SFB^ complex of *P. avium*, a species related to dwarf almond and displaying SI, has been identified and investigated (Matsumoto and Tao, 2016). *SFB* and *S-RNase* alleles of *P. tenella* were investigated in a previous study (Surbanovski et al., 2007), but other genes associated with GSI remain elusive. Here, we chose a putative *P. tenella* accession (ZB1) located in Yumin county, Xinjiang Uygur Autonomous Region of China (45°54′ N, 82°30′E) for subsequent study (**Figure S2**).

The full cDNA sequences of the *PetCUL1* (MH017413) and *PetSSK1* (KT984123) genes were obtained from the NCBI database by informatics, and they encode 733 and 177 amino acids (AA), respectively. To ascertain the sequence accuracy, we also cloned *CUL1* and *SSK1* using pollen cDNA of the ZB1 accession and found that the two cloned genes were identical to *PetCUL1* and *PetSSK1*, respectively, at the nucleotide level. This further confirmed that ZB1 is an accession of *P. tenella*.

The full cDNA sequence of *PetSBP1*, encoding 383 AAs, was cloned using *SBP1* from *P. avium* (PavSBP1, KC244430) as a reference. *SFB*, the pollen S gene determining pollen-part specificity (Yamane et al., 2003), is a single-copy and highly polymorphic gene in the *Prunus* genome (Sassa et al., 2010). Using pollen cDNA of the ZB1 accession, two allelic *SFB* genes were cloned successfully. Through Blast against the NCBI database, we found that the two *SFB* genes were identical to *PetSFB16* (KU167066) and *PetSFB17* (KU167067) at the nucleotide level and encode 381 and 376 AAs (73.5% amino acid identity), respectively.

**Figure 1:**
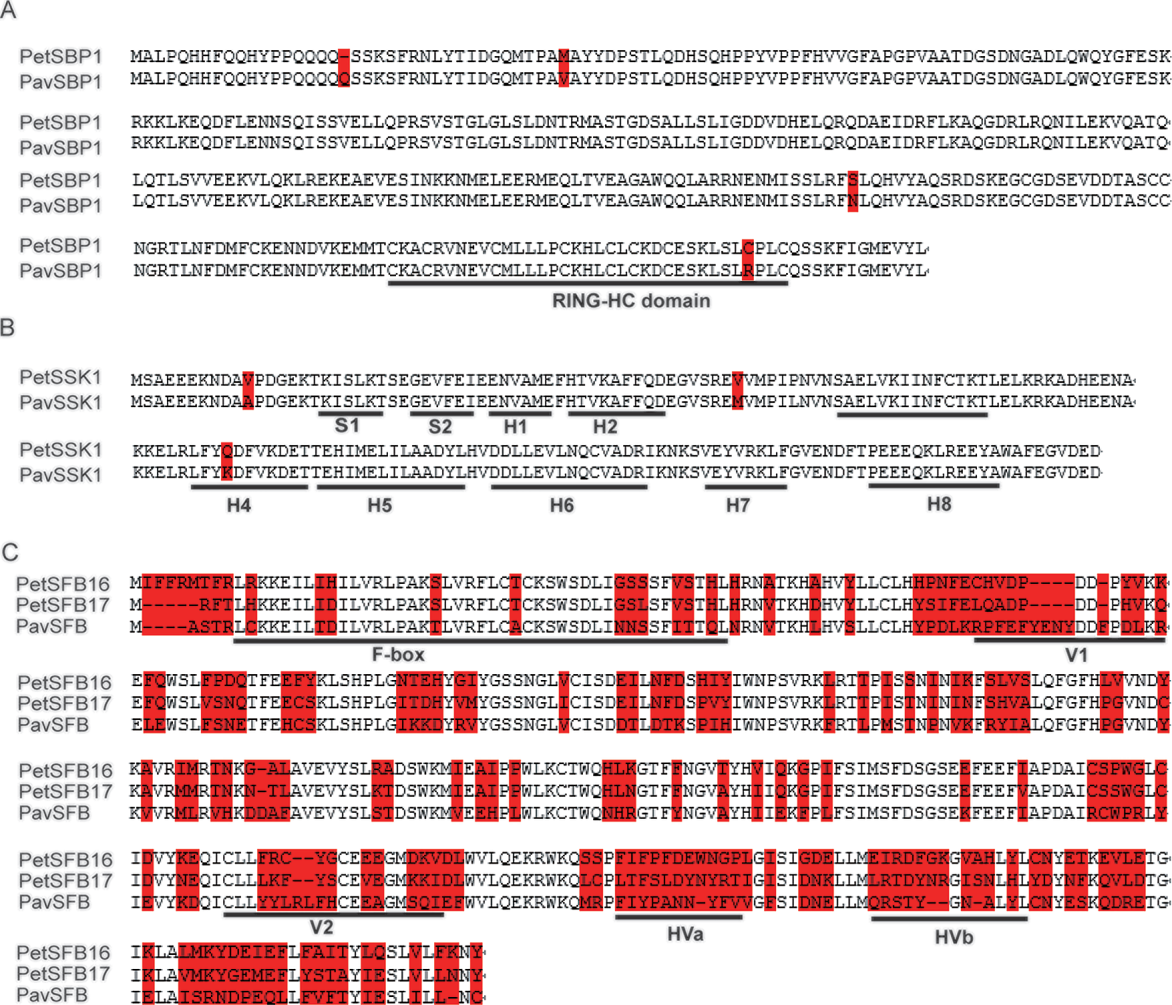
Molecular identification of SBP1 (S-RNase binding protein 1), SSK1 (SLF-interacting SKP1-like1), and SFB (S-haplotype-specific F-box) in the ZB1 accession of *Prunus tenella*. **(A)** Alignments of amino acid (AA) sequences for PetSBP1 and PavSBP1. The RING-HC domain is *underlined*, according to a previous study (Sims and Ordanic, 2001). **(B)** Secondary structures (S, β-sheet; H, α-helix) are *underlined*, according to a previous study (Huang et al., 2006). **(C)** Alignments of AA sequences for PetSFB16, PetSFB17, and PavSFB. Hypervariable (HVa, HVb) and variable (V1, V2) regions and the F-box are *underlined*, according to a previous study (Yamane et al., 2003). *Red shading* indicates variant AAs.

To explore the identities of these homologous genes between *P. tenella* and *P. avium*, we carried out alignments of the deduced amino acid sequences. The identities for *CUL1*, *SBP1*, and *SSK1* between the two species were 99.6%, 98.8%, and 98.3%, respectively (**Figure S3** and **Figures 1A, B**). These high identities suggest that *PetCUL1*, *PetSBP1*, and *PetSSK1* might be orthologous to *PavCUL1*, *PavSBP1*, and *PavSSK1*, respectively. Moreover, we obtained some homologous genes for *CUL1*, *SBP1*, and *SSK1* from the NCBI database (**Table S2**). The results of alignments indicated that these homologous genes were highly conserved at the AA level, and the important domains could be identified (data not shown). For example, the reported RING-HC domains (Sims and Ordanic, 2001) could be found in all analyzed SBP1 proteins, and the reported secondary structures (Hua and Kao, 2006) were successfully identified in all SSK1 proteins. Phylogenetic evolution analyses were carried out (**Figures S4A–C**). The results indicated that *PetCUL1*, *PetSBP1*, and *PetSSK1* were most related to *CUL1*, *SBP1*, and *SSK1* from other *Prunus* species respectively, and the phylogenetic relationships were generally congruent with the taxonomic relationships of these species. It suggested that the three components of the SCF complex might have essential function and be under purifying selection during the evolutionary process.

The identities between PetSFB16 and PavSFB and between PetSFB17 and PavSFB were 56.1% and 60.1%, respectively, and they all contained F-box, hypervariable (HVa, HVb), and variable (V1, V2) regions (**Figure 1C**), suggesting that both *PetSFB16* and *PetSFB17* might be orthologous to *PavSFB*. In addition, more homologous *SFB* genes from *Prunus* species were obtained by informatics (**Table S2**). The results of alignments revealed that these homologous genes were not conserved at the AA level, but the F-box, hypervariable, and variable regions could be found in all analyzed SFB proteins (data not shown). The phylogenetic relationships were generated on the basis of the deduced AA sequences (**Figure S4D**). Interestingly, PetSFB16 was most related to *Prunus speciosa* SFB1 and *Prunus salicina* SFBa, while PetSFB17 was highly similar to *P. avium* SFB13, indicating the incongruence between the phylogenetic and taxonomic relationships. It suggested that the SI function could tolerate a considerable number of mutations in SFB protein sequences without breakdown, and *SFB* genes might be under adaptive selection during the evolutionary process.

### Expression Analysis of *SBP1*, *SSK1*, *CUL1*, and *SFB* Genes

**Figure 2:**
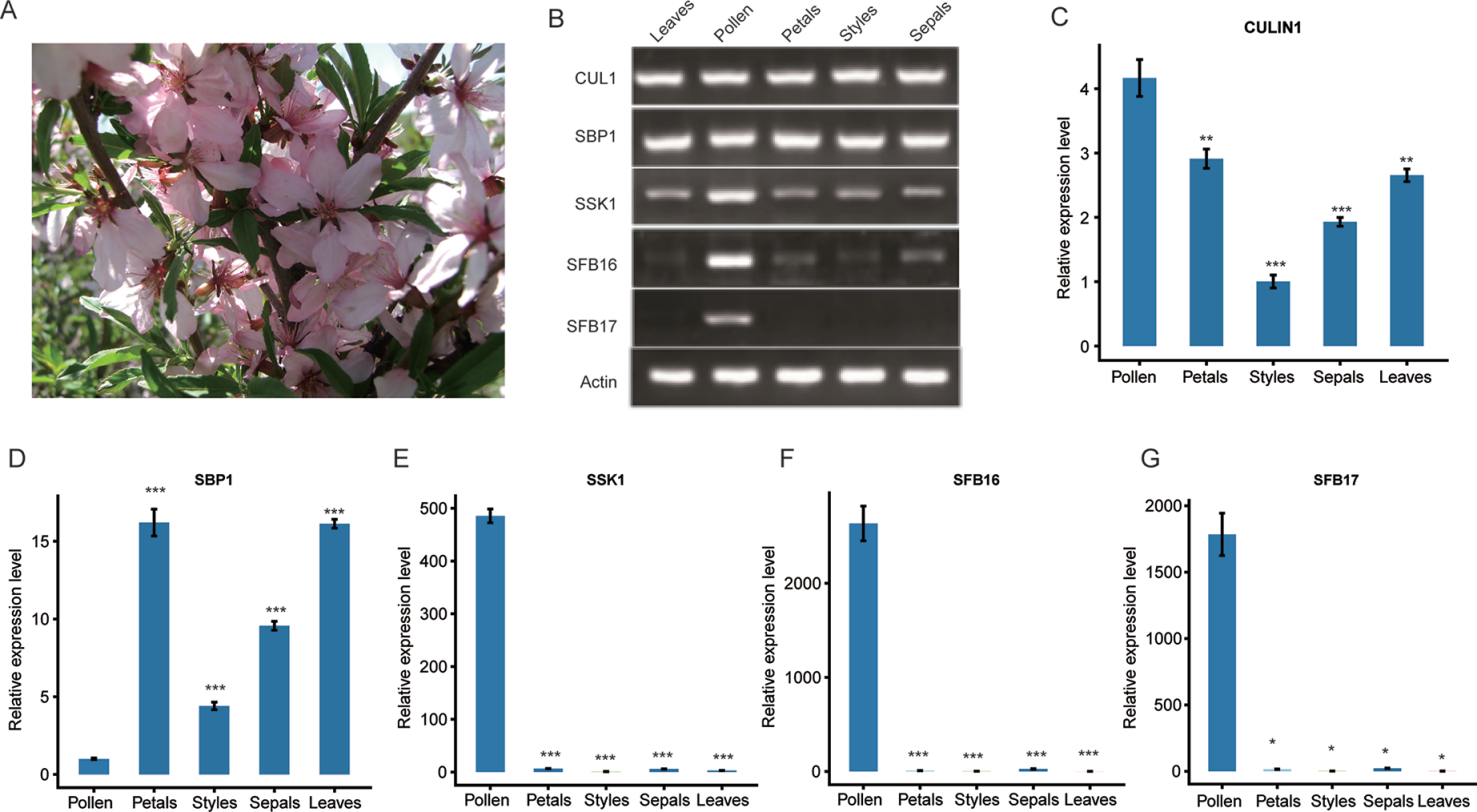
Expression analysis of the *SBP1*, *SSK1*, *CUL1*, *SFB16*, and *SFB17* genes in five organs from wild dwarf almond (*Prunus tenella*). **(A)** Leaves and flowers from an accession (ZB1) of wild dwarf almond. **(B)** The expression patterns of *PetSBP1*, *PetSSK1*, *PetCUL1*, *PetSFB16*, and *PetSFB17* were examined by RT-PCR. Total RNAs were extracted from different organs and used as templates for cDNA synthesis and RT-PCR. **(C**–**G)** mRNA expression levels of *PetSBP1*, *PetSSK1*, *PetCUL1*, *PetSFB16*, and *PetSFB17* were detected by qRT-PCR, respectively. Normalization was achieved using the *Actin* gene. Data are presented as the mean ± standard deviation and the experiment was carried out in triplicate. Statistically significant differences between means were achieved by Student’s *t* test (paired). *P < 0.05, **P < 0.01, and *** *P* < 0.001.

To detect gene expression levels, we collected leaves and floral organs of ZB1 accession in the spring (**Figure 2A**) and prepared the cDNAs accordingly. Reverse-transcription PCR (RT-PCR) experiments indicated that *PetCUL1* and *PetSBP1* were expressed in leaves, styles, pollen, petals, and sepals (**Figure 2B**), indicating that they might be general proteins functioning in numerous pathways. Both *PetSSK1* and *PetSFB16* were expressed strongly in pollen, but very weakly in other floral organs and leaves (**Figure 2B**). *PetSFB17* was only expressed strongly in pollen, and not at all in leaves or other floral organs (**Figure 2B**). Generally, these results were consistent with previous studies (Yuan et al., 2014). Quantitative real-time PCR (qRT-PCR) experiments showed similar results (**Figures 2C–G**).

### Investigation of Interactions Between PetCUL1 and PetSSK1

In sweet cherry (*P. avium*), Y2H and pull-down assays confirmed that PavSSK1 interacts with PavCUL1 (Matsumoto et al., 2012). Because SSK1 and CUL1 between *P. tenella* and *P. avium* showed more than 98% identity, we postulated that PetSSK1 could also interact with PetCUL1 *in vitro*.

**Figure 3:**
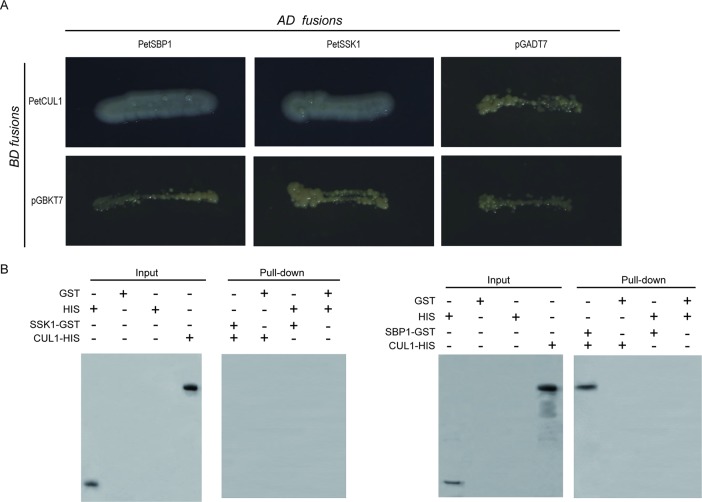
Interactions between PetSSK1 and PetCUL1 and between PetSBP1 and PetCUL1. **(A)** Yeast two-hybrid (Y2H) analysis to investigate interactions between PetSBP1 and PetCUL1 and between PetSSK1 and PetCUL1. The indicated combinations of bait (BD fusion) and prey (AD fusion) constructs were co-transformed into the yeast reporter strain AH109. These transformants were streaked on selective medium SD/-Ade-His-Leu-Trp and detected for growth. Both pGADT7 and pGBKT7 were used as negative controls. Plates were photographed after 5 days of incubation at 30°C. **(B)** GST pull-down assays to investigate interactions between PetSBP1 and PetCUL1 and between PetSSK1 and PetCUL1. Bound proteins were examined using anti-His antibody. The negative control was GST. There were three replications for the experiments of Y2H and GST pull-down array.

In order to test this hypothesis, the full *PetCUL1* coding sequence (CDS) was placed into the pGBKT7 vector, while the complete CDS of *PetSSK1* was placed into the pGADT7 vector. The two vectors were co-transformed into the yeast reporter strain AH109, and the results exhibited that PetSSK1 could interact with PetCUL1 (**Figure 3A**).

Moreover, the Y2H assays were also performed in ProQuest Two-Hybrid System. The intact CDS of *PetCUL1* was introduced into the pDEST32 vector, while *PetSSK1* CDS was placed into the pDEST22 vector. The two vectors were co-transformed into the yeast reporter strain MaV203, and the results also indicated that PetSSK1 could interact with PetCUL1 (**Figure S5A**).

To further confirm the interaction between PetSSK1 and PetCUL1, *PetSSK1* and *PetCUL1* were cloned into the pGEX-4T-1 and pET-32a vectors to express glutathione S-transferase (GST)- and His_6_-tagged fusion proteins, respectively. Subsequently, pull-down assays were carried out (**Figure 3B**). To our surprise, PetSSK1 could not bind with PetCUL1, suggesting that there might be only a weak interaction between PetSSK1 and PetCUL1.

### Investigation of Interactions Between PetCUL1 and PetSBP1

In apple (*M. domestica*), there is conflicting evidence for the interaction between SBP1 and CUL1. Yuan et al. found no interaction between the two proteins by Y2H and pull-down assays (Yuan et al., 2014), while Minamikawa et al. indicated that MdSBP1 could bind to MdCUL1 through pull-down assays (Minamikawa et al., 2014). To our knowledge, there have been no studies on the interaction between CUL1 and SBP1 in *Prunus* species. Although the amino acid identities for CUL1 and SBP1 between *P. tenella* and *M. domestica* were more than 90%, it was still hard to conclude whether PetCUL1 interacted with PetSBP1 or not.

To explore the putative PetSBP1–PetCUL1 interaction, the full-length PetSBP1 CDS was cloned into the pGADT7 vector and then co-transformed into AH109 with the pGBKT7 vector containing PetCUL1. The results revealed that PetSBP1 could interact with PetCUL1. In addition, the complete PetSBP1 CDS was introduced into the pDEST22 vector and co-transformed into MaV203 with the pDEST32 vector containing PetCUL1. The results also indicated that PetSBP1 could bind with PetCUL1 (**Figure S5A**).

Moreover, PetSBP1 was placed into the pGEX-4T-1 vector to express a GST-tagged fusion protein. A pull-down assay carried out between PetSBP1 and the His_6_-tagged PetCUL1 (**Figure 3B**) also confirmed that PetSBP1 could interact with PetCUL1, suggesting that PetSBP1 might be a component of the SCF complex.

### Investigation of Interactions Between PetSSK1 and PetSFBs

In *P. avium*, PavSSK1 interacts with PavSFB in Y2H and pull-down assays (Matsumoto et al., 2012). PetSSK1 is highly homologous to PavSSK1 (**Figure 1B**), but the identities between PetSFB16 and PavSFB and between PetSFB17 and PavSFB were <70% at the AA level. There were many variant AAs in the F-box, hypervariable (HVa, HVb), and variable (V1, V2) regions (**Figure 1C**), so it was uncertain whether PetSSK1 could interact with PetSFBs or not.

**Figure 4:**
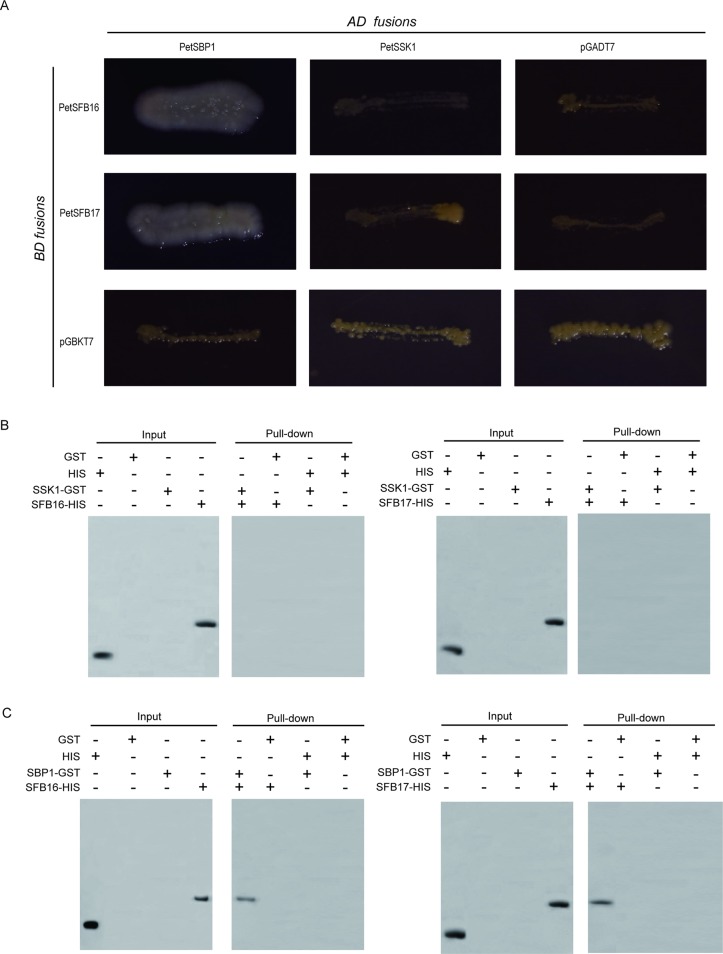
Interactions between PetSBP1 and PetSFBs and between PetSSK1 and PetSFBs (SFB16 and SFB17). **(A)** Yeast two-hybrid (Y2H) analysis to investigate interactions between PetSBP1 and PetSFBs and between PetSSK1 and PetSFBs. The indicated combinations of bait (BD fusion) and prey (AD fusion) constructs were introduced into the yeast reporter strain AH109. All transformants were streaked on selective medium SD/-Ade-His-Leu-Trp and tested for growth. pGADT7 and pGBKT7 were used as negative controls. Plates were photographed after 5 days of incubation at 30°C. **(B)** GST pull-down assays to explore interactions between PetSSK1 and PetSFBs. **(C)** GST pull-down assays to explore interactions between PetSBP1 and PetSFBs. Using an anti-His antibody, the bound proteins were examined accordingly. GST was used as a negative control. There were three replications for the Y2H and GST pull-down experiments.

The intact CDSs of *PetSFB16* and *PetSFB17* were placed into pGBKT7 vectors, respectively. These two vectors were then co-transformed, respectively, into AH109 with the pGADT7 vector containing PetSSK1. To our surprise, the results indicated that neither PetSFB16 nor PetSFB17 could bind to PetSSK1 (**Figure 4A**). Also, the integral CDSs of *PetSFB16* and *PetSFB17* were cloned into pDEST32 vectors, respectively. These two vectors were then co-transformed, respectively, into MaV203 with the pDEST22 vector containing PetSSK1. The results also revealed that both PetSFB16 and PetSFB17 could not bind to PetSSK1 (**Figure S5B**).

Moreover, to produce His-tagged fusion proteins, full-length CDSs of PetSFB16 and PetSFB17 were placed into the pET-32a vector, respectively. Pull-down assays were carried out between PetSSK1 and PetSFB16 and between PetSSK1 and PetSFB17, with the results further confirming that there were no interactions between PetSSK1 and PetSFBs (**Figure 4B**), suggesting that the canonical SSK1-containing SCF^SFB^ complex might not exist in the ZB1 accession.

### Investigation of Interactions Between PetSBP1 and PetSFBs

Because there were no interactions between PetSSK1 and PetSFBs, it is reasonable that there is not a canonical SSK1-containing SCF^SFB^ complex in *P. tenella*. The studies above confirmed that PetCUL1 could interact with PetSBP1, so we speculate that there might be a non-canonical SBP1-containing SCF^SFB^ complex.

To confirm this hypothesis, Y2H assays were also carried out, revealing that both PetSFB16 and PetSFB17 could bind to PetSBP1 (**Figure 4A** and **Figure S5B**). Furthermore, the results of pull-down assays indicated that PetSFB16 and PetSFB17 could bind to PetSBP1 (**Figure 4C**), suggesting a novel SBP1-containing SCF^SFB^ complex in *P. tenella*.

## Discussion

SI allows the pistil to deny genetically related pollen and promotes outcrossing in flowering plants, which contributes to genetic diversity (Fujii et al., 2016). However, it has also hindered inbred lines of crops. For example, SI is a main obstacle for stable fruit production in Rosaceae trees (Sassa, 2016). Therefore, the study of the SI of crops is of importance to provide the basis to generate high-quality self-compatible (SC) cultivars and/or develop techniques to overcome SI. For example, SC diploid potatoes were created by knocking out the SI gene S-RNase using the CRISPR–Cas9 system (Ye et al., 2018).

SI has evolved at least 35 times independently in different flowering plant lineages (Aguiar et al., 2015), so there might be different SI systems in different species. Until now, two alternative models have been proposed to explicate the biochemical mechanism of S-RNase-based SI in Solanaceae, Plantaginaceae, and Rosaceae. The degradation model is involved in a putative canonical SCF^SLF/SFB^ complex containing SSK, SLF/SFB, and CUL1, or a non-canonical SCF^SLF/SFB^ complex embodying SBP1, SLF/SFB, and CUL1, which are proposed to specifically act upon non-self S-RNase for ubiquitination and subsequent degradation through the ubiquitin proteasome pathway, resulting in non-self-pollen acceptance while maintaining self S-RNase intact to perform its cytotoxic activity, leading to self-pollen rejection (Zhang et al., 2009; Meng et al., 2011). To date, canonical SSK-containing SCF^SLF/SFB^ complexes have been identified in multiple species, while non-canonical SBP1-containing SCF^SLF/SFB^ complexes have only been found in *P. inflata* and *M. domestica*.

The wild dwarf almond, an endangered wild relative of cultivated almond, is of potential interest for breeding drought-tolerant rootstocks resistant to extreme temperatures and high oil contents of seeds (Surbanovski et al., 2007; Matthaus and Ozcan, 2014) and may have great potential in almond crop breeding. In previous studies, GSI status was identified in *P. tenella* (Surbanovski et al., 2007). Although the genes of the S loci were cloned and analyzed, other genes associated with GSI remained uninvestigated. In the current study, these components of the SCF complex, which serves as an E3 ubiquitin ligase on non-self S-RNase, including CUL1, SSK1, SBP1, and SFBs, were identified in a *P. tenella* accession (ZB1), and interactions among them were investigated through Y2H and pull-down assays. The results suggested that a novel SBP1-containing SCF^SFB^ complex might exist in *Prunus* species. To our knowledge, this is the first evidence for *Prunus* species. It could be concluded that the SBP1-containing SCF^SLF/SFB^ complex should originate independently in *P. inflata*, *M. domestica*, and *P. tenella*, suggesting the evolutionary convergence of a novel molecular mechanism of gametophytic SI. Maybe, the SBP1-containing SCF^SLF/SFB^ complexes can be found in more species in the future.

Why does PetSBP1 have a function in bridging CUL1 and F-box? In this study, using PetSFB16, PetSFB 17, and PetCUL1 as query proteins, prediction for potential binding sites for PetSBP1 was carried out through protein–protein docking strategies. As many as 82 putative binding sites were found (**Figure S6**), accounting for 25% full residues of PetSBP1, suggesting that PetSBP1 might have specific bonds or structures suitable for interacting with PetCUL1 and PetSFBs.

Although there might be no canonical SSK1-containing SCF^SFB^ complex in the ZB1 accession, we cannot exclude that this putative complex would be found in other *P. tenella* accessions. Although PetSSK1 could not interact with PetSFB16 or PetSFB17, it could bind to PetCUL1. It is possible that polymorphism of SFBs could allow some PetSFBs to bind to both PetSSK1 and PetSBP1 in some *P. tenella* accessions, while other PetSFBs can only interact with PetSSK1. If enough *P. tenella* accessions can be collected and investigated, the canonical SSK1-containing SCF^SLF/SFB^ complex may be found.

It is easy to neglect the SBP1-containing SCF^SLF/SFB^ complex if the SSK-containing SCF^SLF/SFB^ complex has been identified in a species. We should pay attention to both SBP1- and SSK1-containing SCF^SLF/SFB^ complexes in future studies.

## Materials and Methods

### Plant Materials

The accession (ZB1) of *P. tenella* used in this study is located in Yumin county, Xinjiang Uygur Autonomous Region of China (45°54′ N, 82°30′ E) (**Table S1** and **Figure S2**). Leaves and floral organs (pollen, petals, styles, and sepals) of the ZB1 accession were collected in the spring, frozen in liquid nitrogen, and stored at −80°C for later use.

### RNA Extraction and cDNA Synthesis

Total RNA of leaves and floral organs was extracted using a Plant RNA EASYspinPlus Kit (Aidlab, Peking, China) according to the instructions of the manufacturer. To remove DNA, the RNA was treated with RQ1 DNase (Promega, Madison, WI, USA) for 20 min at 37°C. The quality and quantity of the purified RNA were checked by measuring the absorbance at 260 and 280 nm (*A*_260_ and *A*_280_) using a SmartSpec Plus Spectrophotometer (Bio-Rad Laboratories, Inc., Hercules, CA, USA). RNA integrity was further verified by electrophoresis using a 1.5% agarose gel. All RNA samples were stored at −80°C for future use.

Reverse transcription reactions were performed at 50°C for 1 h and comprised 20 U RNase inhibitor (Invitrogen, Carlsbad, CA, USA), 200 U superscript II (Invitrogen), 3 µg total RNA, 500 ng random hexamers, and some buffers.

### Cloning the *CUL1*, *SSK1*, *SBP1*, and *SFBs* of ZB1 Accession

According to the full cDNA sequences of the *PetCUL1* (MH017413) and *PetSSK1* (KT984123) genes from the NCBI database, we designed primers and cloned the CDSs of *CUL1* and *SSK1* of ZB1 accession successfully by PCR experiments.

To clone the *SBP1* gene of ZB1 accession, two degenerate primers were designed according to homologous sequences (**Table S2**): SBP1-F: 5′-THG TNG GVT TTG CNC CNG GTC C-3′ (forward) and SBP1-R: 5′-AGR CAR AGA TGC TTA CAA GG (reverse). The PCR experiment was carried out using the cDNA of leaves. The amplified fragments were sequenced and found to be homologous to PavSBP1 by alignment analysis. On the basis of the partial sequence of PetSBP1, the 3′ and 5′ ends were acquired by rapid amplification of cDNA ends (RACE) methods using gene-specific primers and adapter primers.

In order to clone *SFB* genes of ZB1 accession, two degenerate primers were designed according to homologous sequences (**Table S2**): SFB-F: 5′-GAA AWC KTA ATC GAC ATC CTM GTA AG-3′ (forward) and SFB-R: 5′-CAM RAA TTC GAT TTC GYC ATA TTT C-3′ (reverse). The PCR experiment was performed using the cDNA of pollens. The amplified fragments were cloned and sequenced, and two different nucleotide sequences were obtained. Subsequently, it revealed that the two nucleotide sequences were identical to *PetSFB16* (KU167066) and *PetSFB17* (KU167067), respectively, through the Blast method. So, we designed primers and cloned CDSs of *SFB*s of ZB1 accession according to *PetSFB16* and *PetSFB17*.

### RT-PCR and qRT-PCR

The expression levels of *SBP1*, *SSK1*, *CUL1*, *SFB16*, and *SFB17* were detected by RT-PCR and qRT-PCR. The *Actin* gene of wild dwarf almond was used as a control. Specific primers were designed according to cDNA sequences. Primer sequences were as follows: *SBP1*: 5′-AGA TCA GTC TCC ACG GGC T-3′ (forward), 5′-CGC AAT CGG TCA CCC TGA G-3′ (reverse); *SSK1*: 5′-ATC CCC AAC GTC AAC AGC G-3′ (forward), 5′-AGC TCC ATG ATG TGC TCG G-3′ (reverse); *CULIN1*: 5′-ACC TGC TTC CGT GAT TTG GT-3′ (forward), 5′-AGC TTC CAT GTG TCC CAT CC-3′ (reverse); *SFB16*: 5′-AGA ACC ACT CCA ATC AGC AGT-3′ (forward), 5′-GGC ACC CTT GTT TGT ACG C-3′ (reverse); *SFB17*: 5′-AGG CTG TAA GGA TGA TGC GT-3′ (forward), 5′-GGA CCT TTC TGA ATG ACG TGG-3′ (reverse); *Actin*: 5′-TCC TGA AGA GCA CCC AGT TC-3′ (forward), 5′-TGG CAA CAT ACA TAG CAG GC-3′ (reverse).

In RT-PCR experiments, the PCR amplification conditions consisted of denaturing at 95°C for 3 min, 34 cycles of denaturing at 95°C for 45 s, annealing at 58°C for 50 s, and extension at 72°C for 50 s, followed by a final extension at 72°C for 5 min.

qRT-PCR was carried out on a Bio-Rad S1000 with Bestar SYBR GreenRT-PCR Master Mix (DBI Bioscience, Shanghai, China). PCR was implemented with denaturing at 95°C for 10 min and 38 cycles of denaturing at 95°C for 15 s followed by annealing and extension at 60°C for 1 min. To calculate relative gene expression, we used the Livak and Schmittgen 2^−ΔΔCt^ method (Livak and Schmittgen, 2001), normalized with the reference gene *Actin*. PCR amplifications were conducted in triplicate for each sample.

### Yeast Two-Hybrid Assays

The Y2H assays were firstly carried out in Matchmaker 3 System (BD Biosciences, Erembodegem, Belgium). The pGADT7 vector (Clontech, CA, USA) was digested by *Bam*H I and *Xho* I (Takara, Dalian, China), while the pGBKT7 vector (Clontech) was digested by *Bam*H I and *Pst* I (Takara). Then enzyme-digested vectors were run on 1.0% agarose gel and purified by two 15-min phenol–chloroform (Solarbio, Peking, China) treatments at 4°C.

The full CDS fragments for the *SBP1* and *SSK1* genes from *P. tenella* were amplified by PCR experiments. The PCR products were digested by *Bam*H I and *Xho* I and introduced into the pGADT7 vector, respectively, to express fusion proteins with the GAL4 activation domain. The full CDS fragments for *CUL1*, *SFB16*, and *SFB17* were also were amplified. Then, the PCR products were digested by *Bam*H I and Pst I and placed into pGBKT7, respectively, to form recombinants with the GAL4 DNA binding domain. The primer sequences were as follows: *SBP1*: 5′-CCC GGG TGG GCA TCG ATA CGG GAT CC ATA TGG CTC TTC CTC AAC ACC AC-3′ (forward), 5′-GTA TCT ACG ATT CAT CTG CAG CTC GAG TTA CAA ATA TAC CTC CAT GCC G-3′ (reverse); *SSK1*: 5′-ACC CGG GTG GGC ATC GAT ACG GGA TCC ATA TGT CGG CCG AGG AGG AGA AGA ACG ACG C-3′ (forward), 5′-GTA TCT ACG ATT CAT CTG CAG CTC GAG TCA GTC CTC ATC AAC TCC TTC-3′ (reverse); *CUL1*: 5′-CAT GGA GGC CGA ATT CCC GGG GAT CCG TAT GGA ACG GAA AAT TAT TGA G-3′ (forward), 5′-GTT ATG CTA GTT ATG CGG CCG CTG CAG TCA TGC AAG ATA CTT GAA CAT G-3′ (reverse); *SFB16*: 5′-CAT GGA GGC CGA ATT CCC GGG GAT CCG TAT GAT TTT CTT CAG GAT GAC ATT C-3′ (forward), 5′-GTT ATG CTA GTT ATG CGG CCG CTG CAG TTA ATA ATT CTT GAA TAA AAC-3′ (reverse); *SFB17*: 5′-CAT GGA GGC CGA ATT CCC GGG GAT CCG TTG TAC ATG CAA GTC ATG GAG TG-3′ (forward), 5′-GTT ATG CTA GTT ATG CGG CCG CTG CAG CTA CCC GAT TGT ACG ATT ATA ATA ATC-3′ (reverse). Different AD and BD vectors were co-transformed into yeast strain AH109 and grown on SD/-Ade-His-Leu-Trp medium at 30°C for 3–5 days. Ten independent clones for each combination were streaked on SD/-Ade -His-Leu-Trp medium and grown for 3–4 days at 30°C. There were three replications for each Y2H assay.

The Y2H assays were also performed in ProQuest Two-Hybrid System (Invitrogen). The full CDSs of *SBP1* and *SSK1* were cloned into pDEST22 vectors to generate fusion proteins containing the GAL4 activation domain, and full CDSs for *CUL1*, *SFB16*, and *SFB17* were introduced into pDEST32 vectors for generating fusion proteins with the GAL4 activation domain using the Gateway technology (Invitrogen). The used primers were as follows: *SSK1*: 5′-GAA TCA AAC AAG TTT GTA CAA AAA AAT GTC GGC CGA GGA GGA GAA GAA CGA CGC-3′ (forward), 5′-TCA AAC CAC TTT GTA CAA GAA AGC TGT CAG TCC TCA TCA ACT CCT TC-3′ (reverse); *SBP1*: 5′-GAA TCA AAC AAG TTT GTA CAA AAA AAT GGC TCT TCC TCA ACA CCA C-3′ (forward), 5′-TCA AAC CAC TTT GTA CAA GAA AGC TGT TAC AAA TAT ACC TCC ATG CCG-3′ (reverse); *CUL1*: 5′-GAA TCA AAC AAG TTT GTA CAA AAA AAT GGA ACG GAA AAT TAT TGA G-3′ (forward), 5′-TCA AAC CAC TTT GTA CAA GAA AGC TGT CAT GCA AGA TAC TTG AAC ATG-3′ (reverse); *SFB16*: 5′-GAA TCA AAC AAG TTT GTA CAA AAA AAT GAT TTT CTT CAG GAT GAC ATTC-3′ (forward), 5′-TCA AAC CAC TTT GTA CAA GAA AGC TGT TAA TAA TTC TTG AAT AAA AC-3′ (reverse); *SFB17*: 5′-GAA TCA AAC AAG TTT GTA CAA AAA AAT GAG ATT TAC ACT ACA TAA G-3′ (forward), 5′-TCA AAC CAC TTT GTA CAA GAA AGC TGT TAA TAA TTA TTG AGT AAA AC-3′ (reverse). Different AD and BD vectors were co-transformed into yeast strain MaV203 and grown on SD/-Ade-His-Leu-Trp medium at 30°C for 3–5 days. Six independent clones for each combination were streaked on SD/-Ade-His-Leu-Trp medium and grown for 3–4 days at 30°C. There were three replications for each combination.

### Glutathione S-Transferase Pull-Down Assays

The pGEX-4T-1 (CW biotech, Peking, China) vector was digested by *Bam*H I and *Xho* I (TaKaRa). The full CDS fragments for the *SBP1* and *SSK1* genes were amplified by PCR experiments. Using *Bam*H I and *Xho* I, the PCR products were digested and then cloned into the digested pGEX-4T-1 vector, respectively, to produce GST fusion proteins. For producing His_6_-tagged fusion proteins, the full CDSs of *CUL1* and *SFB17* were cloned into the pET-32a vector (EMD Biosciences, Novagen) at *Bam*H I and *Xho* I (Takara), while the full CDS of *SFB16* was cloned into the pET-32a vector at *Bam*H I and *Hin*d III (Takara). The used primers were as follows: *SBP1*: 5′-CGG GAT CCA TGG CTC TTC CTC AAC ACC ACT TTC-3′ (forward), 5′-CCG CTC GAG CAA ATA TAC CTC CAT GCC G-3′ (reverse); *SSK1*: 5′-CGG GAT CCA TGT CGG CCG AGG AGG AGA AGA AC-3′ (forward), 5′-CCG CTC GAG GTC CTC ATC AAC TCC TTC-3′ (reverse); *CUL1*: 5′-CCG CTC GAG ATG GAA CGG AAA ATT ATT GAG-3′ (forward), 5′-TTA CGG GAT CCT GCA AGA TAC TTG AAC AT-3′ (reverse); *SFB16*: 5′-CGG GAT CCA TGA TTT TCT TCA GGA TGA CAT TC-3′ (forward), 5′-CCG CTC GAG ATA ATT CTT GAA TAA AAC CAA AC-3′ (reverse); *SFB17*: 5′-CGG GAT CCA TGA ATT TCG ATA GTC CTA TAC AC-3′ (forward), 5′-CCG CTC GAG CCC GAT TGT ACG ATT ATA ATA ATC-3′ (reverse).

All the constructed vectors were transformed into *Escherichia coli* strain (BL21), and the transformed cells were cultured in LB medium containing 60 µg/ml ampicillin at 16°C with shaking at 220 rpm. When the OD_600_ achieved 0.6, isopropyl β-D-1-thiogalactopyranoside (IPTG) was added into the suspension culture to induce protein expression, with the conditions being 22°C for 14 h and 0.2 mM IPTG.

To gather bacteria, centrifugation was carried out, and a PBS buffer was used for suspending the bacteria. Subsequently, an ultrasonic cell disruptor was set at 200 W for crushing, and the ultrasonic frequency worked for 10 s, resting for 8 s for 30 min. The GST-tagged fusion proteins were purified using Glutathione Sepharose 4B (Hyclone, GE Healthcare Life Sciences, Logan, UT, USA) and eluted with buffer comprising 25 mM Tris-HCl (pH 8.0), 150 mM NaCl, 3 mM DTT, and 15 mM reduced glutathione. The His_6_-tagged fusion proteins were purified using Ni-NTA (EMD Biosciences, Novagen) and eluted with buffer comprising 25 mM Tris-HCl (pH 8.0), 150 mM NaCl, and 250 mM imidazole. All the eluted proteins were dialyzed with buffer composed of 25 mM Tris-HCl (pH 8.0), 150 mM NaCl, and 3 mM DTT (wash buffer).

For GST pull-down assays, equal amounts of GST-tagged fusion protein and His_6_-tagged fusion protein were mixed and incubated on ice for 3 h. Subsequently, we loaded the mixture onto Glutathione Sepharose 4B resin columns. After washing five times with buffer, the proteins were eluted with wash buffer supplemented with 15 mM reduced glutathione. The eluates were separated using 12% SDS-PAGE, and then they were transferred into PVDF membranes (Millipore, Billerica, MA, USA) and probed with anti-His (Sigma-Aldrich, Merck KGaA, Darmstadt, Germany). GST and His_6_ from GensCript (Nanjing, China) were used as negative controls. There were three replications for each pull-down assay.

### Sequence Alignment and Phylogenetic Analysis

We obtained the taxonomic relationships of the analyzed species from NCBI (http://www.ncbi.nlm.nih.gov/guide/taxonomy). Nucleotide and AA sequences were aligned using the Clustal W program with the system’s default parameters (Thompson et al., 1994). The phylogenetic trees were constructed using MEGA 7.0 (Kumar et al., 2016). To test the validity of the choice for tree models, the ProtTest 2.4 server (http://darwin.uvigo.es/software/prottest2_server.html) was applied. The tree was reconstructed using a neighbor-joining (NJ) method on the basis of the maximum composite likelihood model. The phylogeny was rooted at midpoint and the confidence levels of the clusters were evaluated through the bootstrap test (1,000 replicates), with default settings.

In order to predict the protein–protein binding sites (PPBSs), a predictor called IPPBS-PseAAC was used (Jia et al., 2016). The web server of IPPBS-PseAAC can be accessed at http://www.jci-bioinfo.cn/iPPBS-PseAAC. 

### Statistical Analysis

All values were presented as the mean ± standard deviation (SD). To determine the significance of the differences between means, Student’s *t* test was performed. A value *P* < 0.05 was considered to be statistically significant.

## Data Availability Statement

All datasets generated/analyzed for this study are included in the article/**Supplementary Material**.

## Author Contributions

BZ and JW designed and managed the study. BZ, QH, ZY, and AA drafted and revised the manuscript. BZ, YT, XZ, and XM performed the analysis. BZ and JW participated in sample collection and conducted the experiments. All authors read and approved the final manuscript.

## Funding

This work was supported by “The National Natural Science Foundation of China” (grant number 31660557), “Central Finance Forestry Science and Technology Extension Demonstration Project” (grant number 2016TG11), and “The Key Disciplines Project of Horticulture of Xinjiang Uygur Autonomous Region” (grant number 2016-10758-3).

## Conflict of Interest

The authors declare that the research was conducted in the absence of any commercial or financial relationships that could be construed as a potential conflict of interest.

## References

[B1] Aguiar B. Vieira J. Cunha A. E. Fonseca N. A. Iezzoni A. van Nocker S. (2015). Convergent evolution at the gametophytic self-incompatibility system in Malus and Prunus. PLoS. One. 10, e0126138. 10.1371/journal.pone.0126138 25993016 PMC4438004

[B2] Akagi T. Henry I. M. Morimoto T. Tao R. (2016). Insights into the prunus-specific s-rnase-based self-incompatibility system from a genome-wide analysis of the evolutionary radiation of s locus-related f-box genes. Plant. Cell. Physiol. 57, 1281–1294. 10.1093/pcp/pcw077 27081098

[B3] Chen G. Zhang B. Zhao Z. Sui Z. Zhang H. Xue Y. (2010). 'A life or death decision' for pollen tubes in S-RNase-based self-incompatibility. J. Exp. Bot. 61, 2027–2037. 10.1093/jxb/erp381 20042540

[B4] Deshaies R. J. Joazeiro C. A. (2009). RING domain E3 ubiquitin ligases. Annu. Rev. Biochem. 78, 399–434. 10.1146/annurev.biochem.78.101807.093809 19489725

[B5] Fujii S. Kubo K. Takayama S. (2016). Non-self- and self-recognition models in plant self-incompatibility. Nat. plants. 2, 16130. 10.1038/nplants.2016.130 27595657

[B6] Goldberg E. E. Kohn J. R. Lande R. Robertson K. A. Smith S. A. Igic B. (2010). Species selection maintains self-incompatibility. Sci. 330, 493–495. 10.1126/science.1194513 20966249

[B7] Hiscock S. J. Tabah D. A. (2003). The different mechanisms of sporophytic self-incompatibility. Philos. Trans. R. Soc. Lond. B. Biol. Sci. 358, 1037–1045. 10.1098/rstb.2003.1297 12831470 PMC1693206

[B8] Hua Z. Kao T. H. (2006). Identification and characterization of components of a putative petunia S-locus F-box-containing E3 ligase complex involved in S-RNase-based self-incompatibility. Plant. Cell. 18, 2531–2553. 10.1105/tpc.106.041061 17028207 PMC1626602

[B9] Hua Z. Kao T. H. (2008). Identification of major lysine residues of S(3)-RNase of Petunia inflata involved in ubiquitin-26S proteasome-mediated degradation in vitro. Plant. J. 54, 1094–1104. 10.1111/j.1365-313X.2008.03487.x 18346191

[B10] Hua Z. H. Fields A. Kao T. H. (2008). Biochemical models for S-RNase-based self-incompatibility. Mol. Plant. 1, 575–585. 10.1093/mp/ssn032 19825563

[B11] Huang J. Zhao L. Yang Q. Xue Y. (2006). AhSSK1, a novel SKP1-like protein that interacts with the S-locus F-box protein SLF. Plant J. Cell Mol. Boil. 46, 780–793. 10.1111/j.1365-313X.2006.02735.x 16709194

[B12] Igic B. Bohs L. Kohn J. R. (2006). Ancient polymorphism reveals unidirectional breeding system shifts. Proc. Natl. Acad. Sci. U. S. A. 103, 1359–1363. 10.1073/pnas.0506283103 16428289 PMC1360522

[B13] Igic B. Busch J. W. (2013). Is self-fertilization an evolutionary dead end? N. phytol. 198, 386–397. 10.1111/nph.12182 23421594

[B14] Igic B. Kohn J. R. (2001). Evolutionary relationships among self-incompatibility RNases. Proc. Natl. Acad. Sci. U S A. 98, 13167–13171. 10.1073/pnas.231386798 11698683 PMC60842

[B15] Jia J. Liu Z. Xiao X. Liu B. Chou K. C. (2016). Identification of protein-protein binding sites by incorporating the physicochemical properties and stationary wavelet transforms into pseudo amino acid composition. J. Biomol. Struct. Dyn. 34, 1946–1961. 10.1080/07391102.2015.1095116 26375780

[B16] Kumar S. Stecher G. Tamura K. (2016). MEGA7: Molecular Evolutionary Genetics Analysis Version 7.0 for Bigger Datasets. Mol. Biol. Evol. 33, 1870–1874. 10.1093/molbev/msw054 27004904 PMC8210823

[B17] Lai Z. Ma W. Han B. Liang L. Zhang Y. Hong G. (2002). An F-box gene linked to the self-incompatibility (S) locus of Antirrhinum is expressed specifically in pollen and tapetum. Plant. Mol. Biol. 50, 29–42. 10.1023/A:1016050018779 12139007

[B18] Livak K. J. Schmittgen T. D. (2001). Analysis of relative gene expression data using real-time quantitative PCR and the 2(-Delta Delta C(T)) Method. Methods 25, 402–408. 10.1006/meth.2001.1262 11846609

[B19] Matsumoto D. Tao R. (2016). Recognition of a wide-range of S-RNases by S locus F-box like 2, a general-inhibitor candidate in the Prunus-specific S-RNase-based self-incompatibility system. Plant. Mol. Biol. 91, 459–469. 10.1007/s11103-016-0479-2 27071402

[B20] Matsumoto D. Yamane H. Abe K. Tao R. (2012). Identification of a Skp1-like protein interacting with SFB, the pollen S determinant of the gametophytic self-incompatibility in Prunus. Plant. Physiol. 159, 1252–1262. 10.1104/pp.112.197343 22548785 PMC3387707

[B21] Matthaus B. Ozcan M. M. (2014). Fatty acid, tocopherol and squalene contents of Rosaceae seed oils. Bot. Stud. 55, 48. 10.1186/s40529-014-0048-4 28510938 PMC5432826

[B22] Meng X. Sun P. Kao T. H. (2011). S-RNase-based self-incompatibility in Petunia inflata. Ann. bot. 108, 637–646. 10.1093/aob/mcq253 21193481 PMC3170144

[B23] Minamikawa M. F. Koyano R. Kikuchi S. Koba T. Sassa H. (2014). Identification of SFBB-containing canonical and noncanonical SCF complexes in pollen of apple (Malus x domestica). PLoS. One. 9, e97642. 10.1371/journal.pone.0097642 24847858 PMC4029751

[B24] Moon J. Parry G. Estelle M. (2004). The ubiquitin-proteasome pathway and plant development. Plant. Cell. 16, 3181–3195. 10.1105/tpc.104.161220 15579807 PMC535867

[B25] Sassa H. (2016). Molecular mechanism of the S-RNase-based gametophytic self-incompatibility in fruit trees of Rosaceae. Breed. Sci. 66, 116–121. 10.1270/jsbbs.66.116 27069396 PMC4780795

[B26] Sassa H. Kakui H. Minamikawa M. (2010). Pollen-expressed F-box gene family and mechanism of S-RNase-based gametophytic self-incompatibility (GSI) in Rosaceae. Sex. Plant. Reprod. 23, 39–43. 10.1007/s00497-009-0111-6 20165962

[B27] Sijacic P. Wang X. Skirpan A. L. Wang Y. Dowd P. E. McCubbin A. G. (2004). Identification of the pollen determinant of S-RNase-mediated self-incompatibility. Nat. 429, 302–305. 10.1038/nature02523 15152253

[B28] Sims T. L. Ordanic M. (2001). Identification of a S-ribonuclease-binding protein in Petunia hybrida. Plant. mol. biol. 47, 771–783. 10.1023/A:1013639528858 11785938

[B29] Sims T. L. Patel A. Shrestha P. (2010). Protein interactions and subcellular localization in S-RNase-based self-incompatibility. Biochem. Soc. Trans. 38, 622–626. 10.1042/BST0380622 20298232

[B30] Steinbachs J. E. Holsinger K. E. (2002). S-RNase-mediated gametophytic self-incompatibility is ancestral in eudicots. Mol. boil. evol. 19, 825–829. 10.1093/oxfordjournals.molbev.a004139 12032238

[B31] Surbanovski N. Tobutt K. R. Konstantinovic M. Maksimovic V. Sargent D. J. Stevanovic V. (2007). Self-incompatibility of Prunus tenella and evidence that reproductively isolated species of Prunus have different SFB alleles coupled with an identical S-RNase allele. Plant. J. 50, 723–734. 10.1111/j.1365-313X.2007.03085.x 17461794

[B32] Thompson J. D. Higgins D. G. Gibson T. J. (1994). CLUSTAL W: improving the sensitivity of progressive multiple sequence alignment through sequence weighting, position-specific gap penalties and weight matrix choice. Nucleic. Acids. Res. 22, 4673–4680. 10.1093/nar/22.22.4673 7984417 PMC308517

[B33] Tyers M. Jorgensen P. (2000). *Proteolysis and the cell cycle: with this RING I do thee destroy* . Curr Opin Genet Dev 10, 54–64. 10.1016/S0959-437X(99)00049-0 10679394

[B34] Vieira J. Fonseca N. A. Vieira C. P. (2008). An S-RNase-based gametophytic self-incompatibility system evolved only once in eudicots. J. Mol. Evol. 67, 179–190. 10.1007/s00239-008-9137-x 18626680

[B35] Xu C. Li M. Wu J. Guo H. Li Q. Zhang Y. (2013). Identification of a canonical SCF(SLF) complex involved in S-RNase-based self-incompatibility of Pyrus (Rosaceae). Plant. Mol. Biol. 81, 245–257. 10.1007/s11103-012-9995-x 23263858

[B36] Yamane H. Ikeda K. Ushijima K. Sassa H. Tao R. (2003). A pollen-expressed gene for a novel protein with an F-box motif that is very tightly linked to a gene for S-RNase in two species of cherry, Prunus cerasus and P. *avium* . Plant. Cell. Physiol. 44, 764–769. 10.1093/pcp/pcg088 12881505

[B37] Ye M. Peng Z. Tang D. Yang Z. Li D. Xu Y. (2018). Generation of self-compatible diploid potato by knockout of S-RNase. Nat. Plants. 4, 651–654. 10.1038/s41477-018-0218-6 30104651

[B38] Yuan H. Meng D. Gu Z. Li W. Wang A. Yang Q. (2014). A novel gene, MdSSK1, as a component of the SCF complex rather than MdSBP1 can mediate the ubiquitination of S-RNase in apple. J. Exp. Bot. 65, 3121–3131. 10.1093/jxb/eru164 24759884 PMC4071834

[B39] Zhang Y. Zhao Z. Xue Y. (2009). Roles of proteolysis in plant self-incompatibility. Ann. rev. plant. biol. 60, 21–42. 10.1146/annurev.arplant.043008.092108 19575579

[B40] Zhao L. Huang J. Zhao Z. Li Q. Sims T. L. Xue Y. (2010). The Skp1-like protein SSK1 is required for cross-pollen compatibility in S-RNase-based self-incompatibility. Plant. J. 62, 52–63. 10.1111/j.1365-313X.2010.04123.x 20070569

[B41] Zheng N. Schulman B. A. Song L. Miller J. J. Jeffrey P. D. Wang P., (2002). Structure of the Cul1-Rbx1-Skp1-F boxSkp2 SCF ubiquitin ligase complex. Nat. 416, 703–709. 10.1038/416703a 11961546

