# Supplementary material for: Identification of a Novel SBP1-Containing SCFSFB Complex in Wild Dwarf Almond (Prunus tenella)
Source: Front Genet. 2019 Oct 25;10:1019. doi: 10.3389/fgene.2019.01019 (PMC6823244; doi:10.3389/fgene.2019.01019)
Supplement: Supplementary file 1 [file DataSheet_1.docx]

**Supplementary data**

**Identification of a novel SBP1-containing SCF^SFB^ complex in wild dwarf almond (*Prunus tenella*)**

Bin Zeng ^1,2*^, Jianyou Wang ^3^, Qing Hao ^4^, Zhenfan Yu ^1,2^, Ayimaiti Abudukayoumu ^1,2^, Yilian Tang ^1,2^, Xiangfei Zhang ^1,2^, Xinxin Ma ^1,2^

^1^College of Forestry and Horticulture Xinjiang Agricultural University/

^2^ Xinjiang Sub-branch of National Melon and Fruit Improvement Centre, Urumqi 830052;

^3^Xinjiang branch of China Academy of Forestry Sciences, Urumqi 830000;

^4^Institute of Horticultural crops, Xinjiang Academy of Agricultural Sciences, Urumqi 830091;

*Corresponding to: Professor Bin Zeng, Collage of Forestry and Horticultural, Xinjiang Agricultural University, XinJiang Urumqi, 830052, P. R. China.

E-mail*:* [zbxjau@163.com](mailto:zbxjau@163.com), Tel: 0086-991-8787811, Fax: 0086-991-8762363

Table S1. Details of five *Prunus tenella* populations in Xinjiang Uygur Autonomous Region of China.

| **Region (county)** | **Population ID** | **Location** | **Altitude(m)** |
| --- | --- | --- | --- |
| **Yuming** | **YM** | **45^°^54^′^N, 82^°^30^′^E** | **1000-1200** |
| **Tuoli** | **TL** | **46^°^09^′^N, 83^°^33^′^E** | **860-1080** |
| **Tacheng** | **TC** | **47^°^03^′^N, 83^°^01^′^E** | **1000-1115** |
| **Burejing** | **BRJ** | **48^°^11^′^N, 87^°^06^′^E** | **1100-1300** |
| **Habahe** | **HBH** | **48^°^18^′^N, 86^°^34^′^E** | **1060-1110** |

Table S2. The homologous *SSK1*, *SBP1*, *CUL1* and *SFB* genes.

| **Species** | **Gene** | **Accession NO. in NCBI** |
| --- | --- | --- |
| *P. hybrida* | SSK1 | FJ490177 |
| *Prunus avium* | SSK1 | JQ322646 |
| *Malus domestica* | SSK1 | NM_001294364 |
| *Pyrus bretschneideri* | SSK1 | HE802072 |
| *Fragari avesca* | SSK1 | XP_004306569 |
| *Antirrhinum hispanicum* | SSK1 | DQ355479 |
| *Prunus tenella* | SSK1 | KT984123 |
| *Solanum chacoense* | SBP1 | AY545464 |
| *Petunia integrifolia* | SBP1 | DQ250022 |
| *Prunus avium* | SBP1 | KC244430 |
| *Corchorus olitorius* | SBP1 | OMO74713 |
| *Arabidopsis thaliana* | SBP1 | NM_103601 |
| *Malus domestica* | SBP1 | AB763431 |
| *Nicotiana alata* | SBP1 | EU591514 |
| *Elaeis guineensis* | SBP1 | JX438717 |
| *Prunus tenella* | SBP1 | MH512897 |
| *Malus domestica* | CUL1 | NM_001328947 |
| *Prunus avium* | CUL1 | JQ322649 |
| *Prunus tenella* | CUL1 | MH017413 |
| *Pyrus bretschneideri* | CUL1 | XM_009380648 |
| *Prunus mume* | CUL1 | XM_008236791 |
| *Prunus persica* | CUL1 | XM_007198936 |
| *Ziziphus jujuba* | CUL1 | XM_016036467 |
| *Juglans regia* | CUL1 | XM_018970541 |
| *Fragaria vesca* | CUL1 | XM_004309629 |
| *Theobroma cacao* | CUL1 | XM_007009762 |
| *Prunus tenella* | SFB16 | KU167066 |
| *Prunus tenella* | SFB17 | KU167067 |
| *Prunus avium* | SFB | JQ322648 |
| *Prunus mume* | SFB1 | AB101440 |
| *Prunus mume* | SFB7 | AB101441 |
| *Prunus speciosa* | SFB1 | HM347508 |
| *Prunus armeniaca* | SFB1 | AY587563 |
| *Prunus armeniaca* | SFB2 | AY587562 |
| *Prunus cerasus* | SFB26 | EU035977 |
| *Prunus dulcis* | SFBa | AB092966 |
| *Prunus dulcis* | SFBk | AB252408 |
| *Prunus persica* | SFB1 | AB252414 |
| *Prunus persica* | SFB3 | AB537564 |
| *Prunus salicina* | SFBa | AB252410 |
| *Prunus salicina* | SFBe | AB280794 |
| *Prunus avium* | SFB2 | AB111519 |
| *Prunus avium* | SFB7 | EU035976 |
| *Prunus avium* | SFB13 | DQ385844 |

**
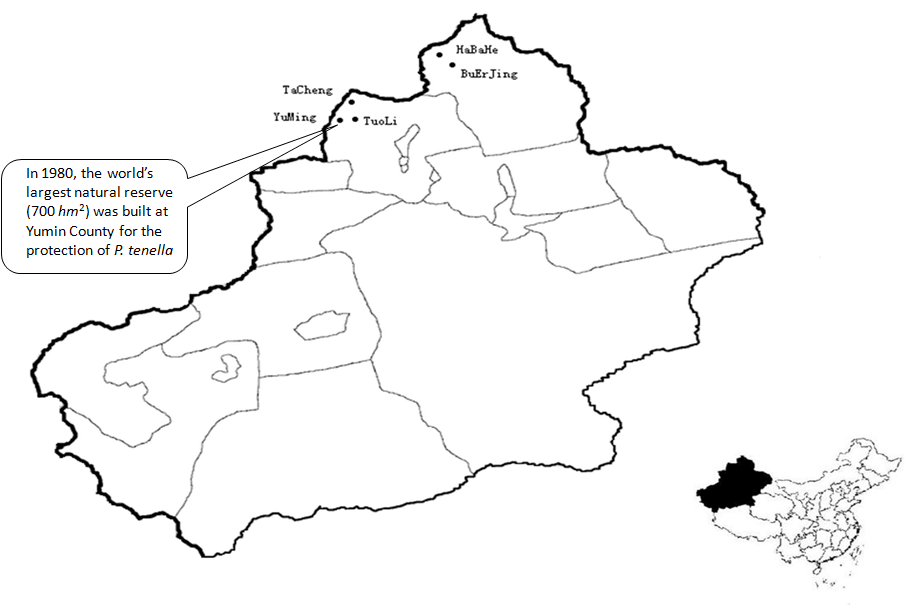
**

**Figure S1.** Natural distributions of five *Prunus tenella* populations in Xinjiang Uygur Autonomous Region of China.


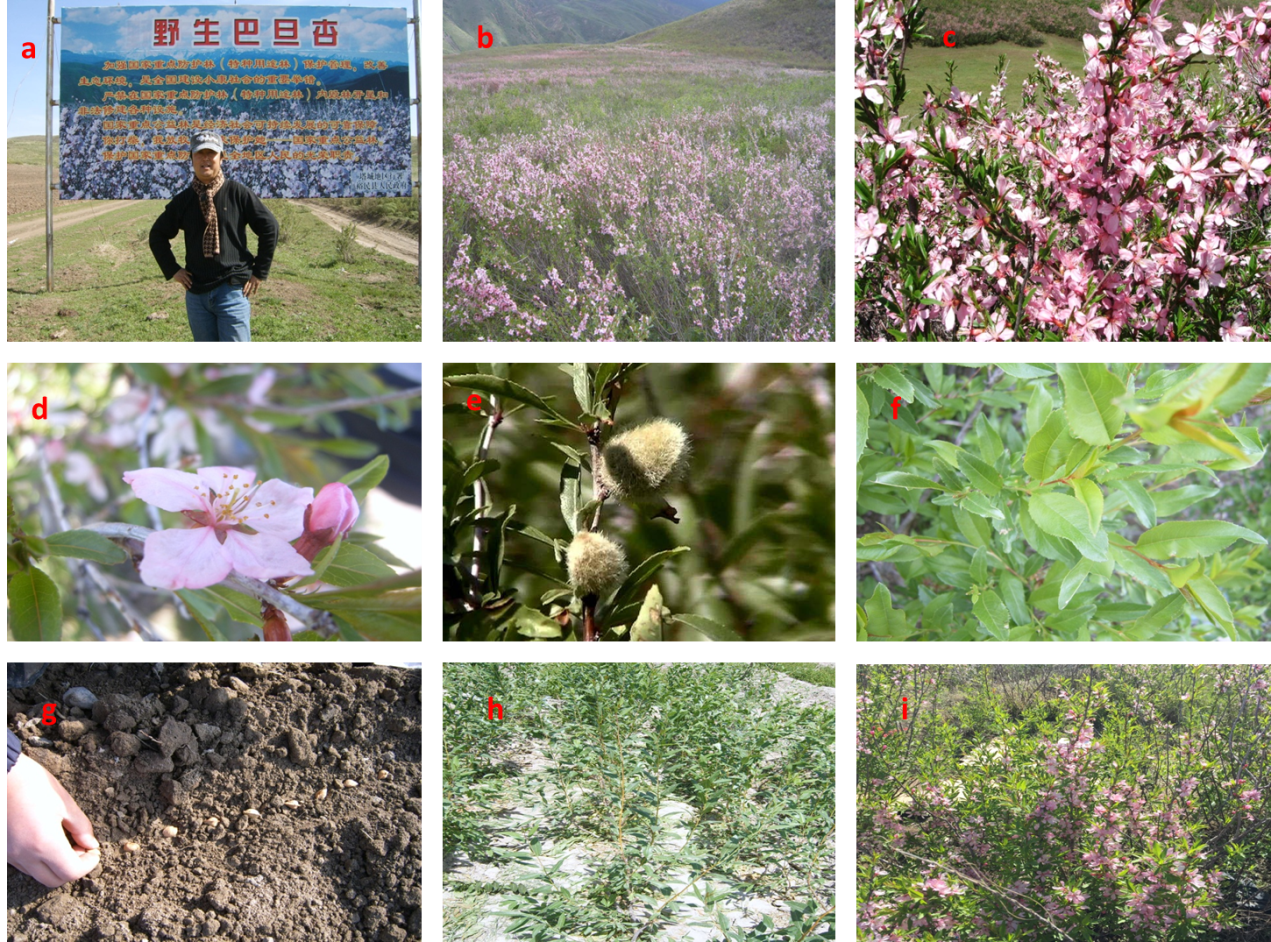


**Figure S2.** The population of *Prunus tenella* in Yumin county, Xinjiang Uygur Autonomous Region of China (45°54' N; 82°30'E).

**
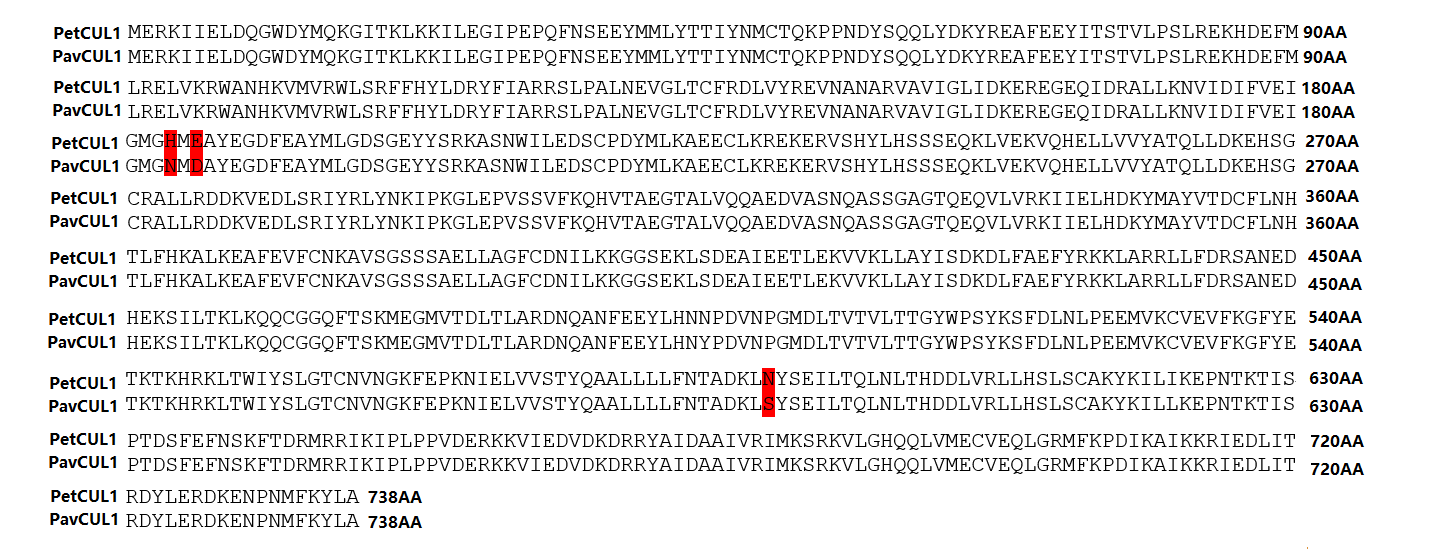
**

**Figure S3.** Alignment of deduced amino acid (AA) sequences for PetCUL1 and PavCUL1. Red shading indicates variant AAs.


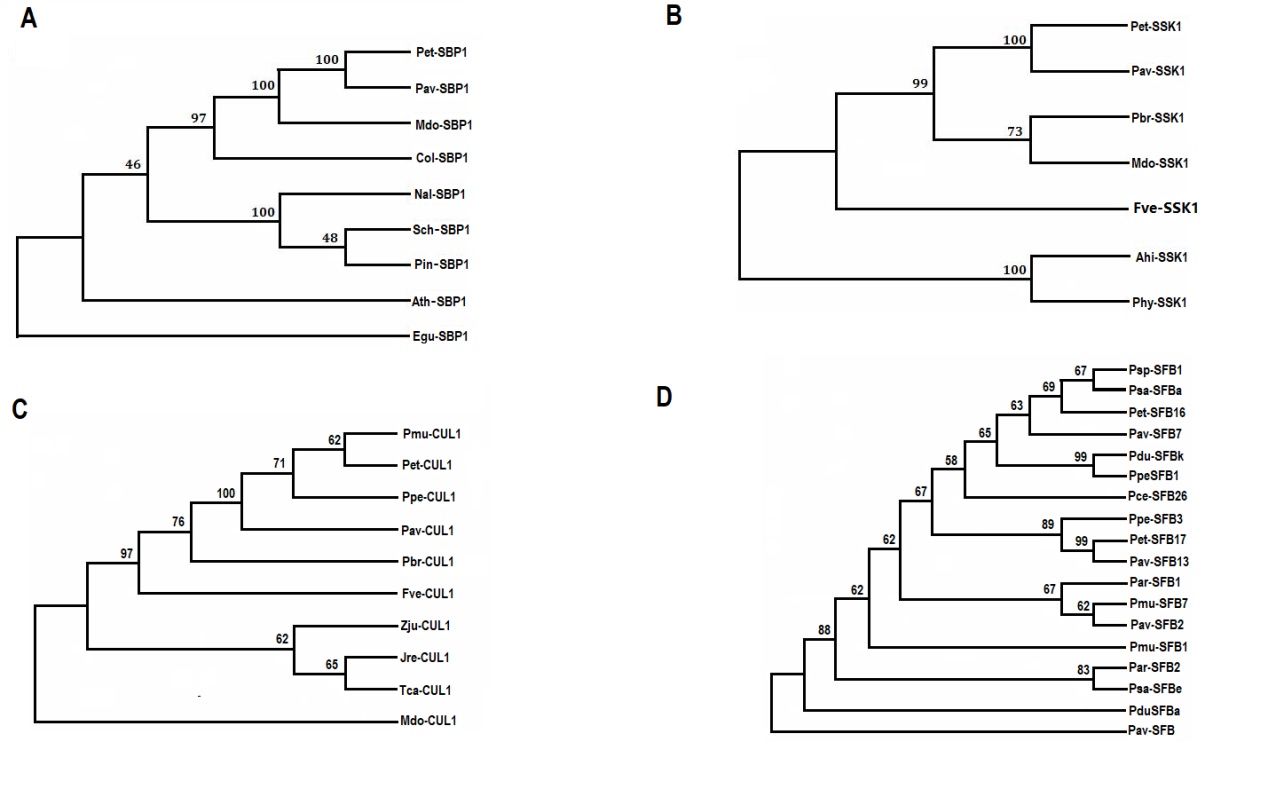


**Figure S4.** Phylogentic evolution analyses for *SSK1*, *SBP1*, *CUL1* and *SFB* genes. The tree was reconstructed on basis of deduced amino acid sequences using a neighbor-joining (NJ) method based on the maximum composite likelihood model. The phylogeny was rooted on midpoint. The confidence levels of the clusters were evaluated by the bootstrap test (1000 replicates) with default settings. These homologous genes were obtained from NCBI database, and the accession numbers were presented in Table S2. Abbreviations: Pet, *Prunus tenella*; Pav, *Prunus avium*; Pmu, *Prunus mume*; Psp, *Prunus speciosa*; Par, *Prunus armeniaca*; Pce, *Prunus cerasus*; Pdu, *Prunus dulcis*; Ppe, *Prunus persica*; Psa, *Prunus salicina*; Mdo, *Malus domestica*; Pbr, *Pyrus bretschneideri*; Sch, *Solanum chacoense*; Zju, *Ziziphus jujube*; Jre, *Juglans regia*; Tca, *Theobroma cacao*; Col, *Corchorus olitorius*; Nal, *Nicotiana alata*; Pin, *Petunia integrifolia*; Ath, *Arabidopsis thaliana*; Egu, *Elaeis guineensis*; Fve, *Fragaria vesca*; Phy, *Petunia hybrida*; Ahi, *Antirrhinum hispanicum*; .


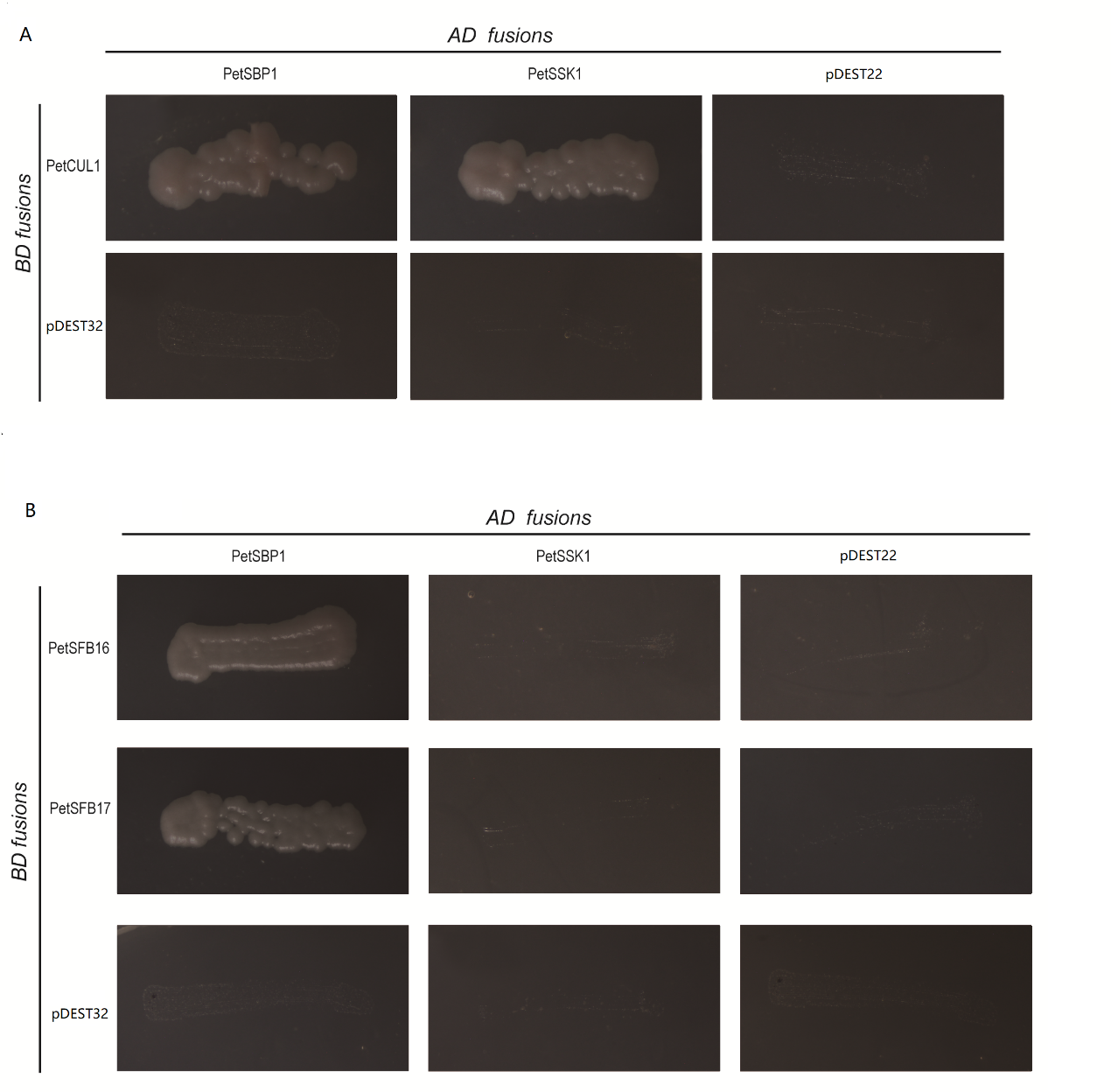


**Figure S5.** Yeast two-hybrid (Y2H) assays were carried out in ProQuest Two-Hybrid System (Invitrogen, Carlsbad, CA). (A) Y2H analysis to investigate interactions between PetSBP1 and PetCUL1 and between PetSSK1 and PetCUL1. (B) Y2H analysis to investigate interactions between PetSBP1 and PetSFBs and between PetSSK1 and PetSFBs. The indicated combinations of bait (BD fusion) and prey (AD fusion) constructs were introduced into the yeast reporter strain MaV203. All transformants were streaked on selective medium SD/-Ade-His-Leu-Trp and examined for growth. Empty vectors pDEST22 and pDEST32 were used as negative controls. Plates were photographed after 5 days of incubation at 30 °C. There were three replications for each Y2H array.

**
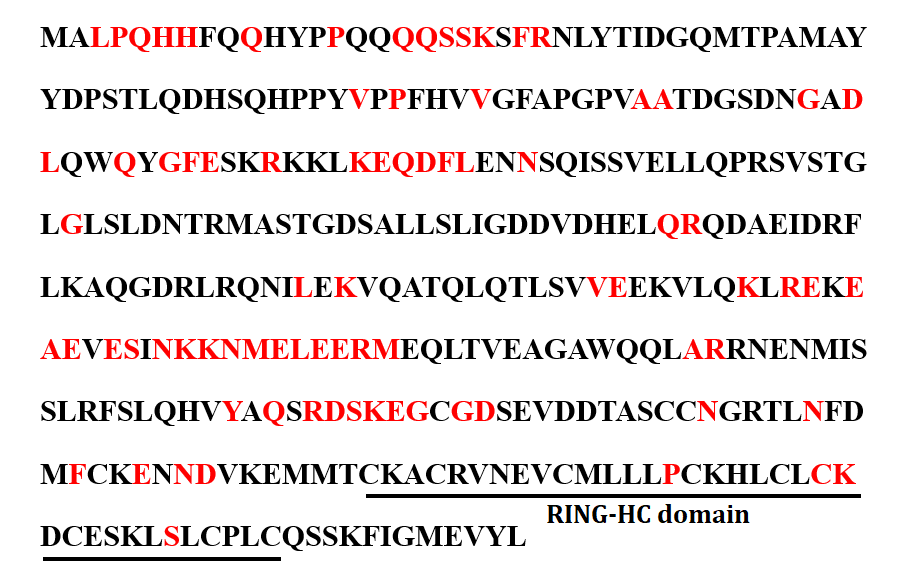
**

**Figure S6.** Prediction of potential binding sites for PetSBP1. PetSFB16, PetSFB 17 and PetCUL1 were used as query proteins through protein-protein docking strategies. In order to predict the protein-protein binding sites (PPBSs), a predictor called IPPBS-PseAAC was used. The web server of IPPBS-PseAAC is accessible at <http://www.jci-bioinfo.cn/iPPBS-PseAAC>. Red amino acids (AAs) indicate potential binding sites.
